# Feasibility Study of HIV Sentinel Surveillance using PMTCT data in Cameroon: from Scientific Success to Programmatic Failure

**DOI:** 10.1186/s12879-016-2119-5

**Published:** 2017-01-03

**Authors:** Serge C. Billong, Jacob Dee, Joseph Fokam, Georges Nguefack-Tsague, Gabriel L. Ekali, Raoul Fodjo, Edith S. Temgoua, Edson-Joan Billong, Samuel M. Sosso, Jembia J. Mosoko, Francisca Monebenimp, Alexis Ndjolo, Anne-Cecile Z-K. Bissek, Omotayo Bolu, Jean-Bosco N. Elat

**Affiliations:** 1 National HIV drug resistance surveillance and prevention Working Group (HIVDR-WG), National AIDS Control Committee, Yaoundé, Cameroon; 2 Faculty of Medicine and Biomedical Sciences (FMBS), University of Yaoundé1, Yaoundé, Cameroon; 3 Central Technical Group, National AIDS Control Committee, Ministry of Public Health, Yaoundé, Cameroon; 4 Centers for Disease Control and Prevention, Division of Global HIV/AIDS, Atlanta, USA; 5 Chantal BIYA International Reference Centre (CIRCB) for research on HIV/AIDS prevention andmanagement, Yaoundé, Cameroon; 6 Chair of Virology, Department of Experimental Medicine and Surgery, Faculty of Medicine and Surgery, University of Rome Tor Vergata, Rome, Italy; 7 Faculty of Medicine, University of Antanarivo, Antananarivo, Madagascar; 8 Centers for Disease Control and Prevention, Division of Global HIV/AIDS, Cameroon Country Office, Yaoundé, Cameroon; 9 Division of Operational Health Research, Ministry of Public Health, Yaoundé, Cameroon

## Abstract

**Background:**

In low-income countries (LICs), HIV sentinel surveillance surveys (HIV-SSS) are recommended in between two demographic and health surveys, due to low-cost than the latter. Using the classical unlinked anonymous testing (UAT), HIV-SSS among pregnant women raised certain ethical and financial challenges. We therefore aimed at evaluating how to use prevention of mother-to-child transmission of HIV (PMTCT) routine data as an alternative approach for HIV-SSS in LICs.

**Methods:**

A survey conducted through 2012 among first antenatal-care attendees (ANC1) in the ten regions of Cameroon. HIV testing was performed at PMTCT clinics as-per the national serial algorithm (rapid test), and PMTCT site laboratory (PMTCT-SL) performances were evaluated by comparison with results of the national reference laboratory (NRL), determined as the reference standard.

**Results:**

Acceptance rate for HIV testing was 99%, for a total of 6521 ANC1 (49 · 3% aged 15–24) enrolled nationwide. Among 6103 eligible ANC1, sensitivity (using NRL testing as the reference standard) was 81 · 2%, ranging from 58 · 8% (South region) to 100% (West region); thus implying that 18 · 8% HIV-infected ANC1 declared HIV-negative at the PMTCT-SL were positive from NRL-results. Specificity was 99 · 3%, without significant disparity across sites. At population-level, this implies that every year in Cameroon, ~2,500 HIV-infected women are wrongly declared seronegative, while ~1,000 are wrongly declared seropositive. Only 44 · 4% (16/36) of evaluated laboratories reached the quality target of 80%.

**Conclusions:**

The study identified weaknesses in routine PMTCT HIV testing. As Cameroon transitions to using routine PMTCT data for HIV-SSS among pregnant women, there is need in optimizing quality system to ensure robust routine HIV testing for programmatic and surveillance purposes.

**Electronic supplementary material:**

The online version of this article (doi:10.1186/s12879-016-2119-5) contains supplementary material, which is available to authorized users.

## Background

Cameroon is a low-income country (LIC) of the central Africa region, with an estimated population of 19 · 4 million inhabitants in 2010 and a generalized epidemiology of human immunodeficiency virus (HIV) in the general population (4 · 3% prevalence) [1, 2]. In a context of generalized HIV epidemic coupled with limited resources, demographic and health surveys (DHS) represent a key component for epidemic surveillance. However, because DHS are of important financial resources for LICs, such surveys are recommended by the world health organization (WHO) on 5 years intervals [3–5]. Of note, the epidemiological surveillance component of HIV, of acquired immunodeficiency syndrome (AIDS) and of sexually transmitted infections (STI) aims at guiding control strategies against the AIDS pandemics, by providing reliable evidence on the burden and the geographical distribution of infection. Data from such surveys are essential for planning, implementation, monitoring and evaluation of preventive, management, and attenuation of the epidemiological impact within specific settings [3].

Between two DHS, the trends of HIV prevalence in the population are assessed through sentinel surveillance surveys (SSS) conducted every 2 years among sub-populations. Particularly, the sub-population of first antenatal care attendees (ANC1) represents a suitable target, as-per WHO guidelines [3]. In this target population, HIV-SSS is based on unlinked anonymous testing (UAT) wherein leftover bloods from pregnant women are tested to determine HIV prevalence in this population. Due to financial challenges in conducting SSS, the frequency of such surveys remains low in several LICs (i.e. only two surveys conducted in Cameroon after 13 years: in 2000 and 2009) [6]. Moreover, HIV-SSS among ANC1, in its classical model of UAT, raises certain ethical concerns in that it does not: (a) obtain informed consent from pregnant women tested for HIV in the context of surveillance, (b) provide them their surveillance HIV test results to those women, and (c) refer them to available HIV care, treatment, and prevention of mother to child transmission (PMTCT) services if surveillance HIV test results are positive [6]. Additionally, this HIV-SSS model also raises challenges in data standardization and in designing reliable epidemiological surveillance models for LICs [3, 6].

As PMTCT programs expand in coverage and capture socio-demographic, syphilis, and HIV testing data similar to those collected by SSS, many countries are transitioning to using routinely collected PMTCT program data to replace UAT-based HIV-SSS. Studies conducted in sub-Saharan Africa (Kenya in 2003, Botswana and Uganda in 2005, Zimbabwe in 2007, Senegal in 2008, and recently Mozambique in 2013) have shown evidence of consistency between HIV-SSS data and routine PMTCT program data. Factors favoring the suitability of PMTCT program data were high ANC coverage, high acceptance in HIV testing, and high-quality data in PMTCT registers and in laboratory records [7–11]. In the same line, India, Thailand and Rwanda used routine PMTCT data for HIV-SSS or for providing epidemiological information to strengthen surveillance [12–15]. Studies conducted in Cameroon in 2003 and in 2007 reported HIV prevalence data from PMTCT programs to be similar to surveillance estimates and potentially reliable for monitoring the epidemic, provided there is good site laboratory performance and high acceptance for HIV testing in ANC [6, 16]. However, Marsh et al.*,* showed in 2010 that there were substantial gaps in the quality of routine data and testing that need to be addressed during transition [17]. In accordance with WHO-guidelines [3], and considering the ongoing program on strengthening laboratories capacity for HIV testing (supported by the Centre for Disease Control and prevention [CDC]), and lessons from previous studies, it was deemed relevant to perform a more comprehensive study to identify routine PMTCT HIV testing quality gaps, and quality strengthening strategies to support transition to a PMTCT-based system of HIV-SSS among pregnant women.

In Cameroon, PMTCT services are available in all 182 health districts and in 81% ANC sites (1674/2067); ANC attendance is 42% and registers are standardized with relevant information for HIV-SSS [18]. Thus, envisaging PMTCT data for estimating HIV prevalence among ANC1 seems rationale [17–20]. We aimed at identifying PMTCT quality gaps and strengthening measures that will ensure a successful transition to using routine PMTCT data for HIV-SSS in Cameroon. Specifically, we aimed at assessing the acceptance rates of HIV testing at ANC clinics, the intrinsic (sensitivity, specificity) and extrinsic (positive predictive value [PPV], negative predictive value [NPV]) performances of HIV testing at PMTCT-site laboratories (SLs) compared to reports from the National Reference Laboratory (NRL), and the laboratory quality system in place.

## Methods

### Study design

A prospective study was conducted through 2012 in the ten regions of Cameroon, covering 20 SSS sites that include 60 SSS routine collection points. PMTCT sites were chosen based on their localization (urban and rural representation for each region of the country), coverage of ANC1 in the catchment area (≥60%, as recommended by the WHO), ANC client volume (ability to enroll ≥300–500 pregnant women at ANC1 during the study period of ≈ 3 months); and presence of ANC and PMTCT services [3]. Urban regions had one site while rural regions had three. In each selected site, pregnant women attending their first ANC (ANC1) aged 15–49 were consecutively enrolled, until achieving the required site sample size (i.e. 300 or 500 ANC1 per site), calculated based on assumed site prevalence and a desired precision of 95% [3].

### Procedure for sample collection and HIV testing

Plasma samples and sociodemographic data were collected without altering the site routine functionality. During the study period, HIV and syphilis screening were proposed to ANC1 attendees according to routine procedure. HIV and syphilis screening were then performed on-site, and only syphilis result was delivered according to the classical SSS procedure [3]. After HIV and syphilis testing on-site at ANC1, residual plasma were transferred into cryotubes that were marked with the ID code, and stored at 0°–8 °C. Plasma and form, linked by the ID code, were then permanently delinked from corresponding woman. Only delinked samples were then taken to the central level (NRL) as-per universal standards for transport of plasma specimens [19].

**Fig. 1:**
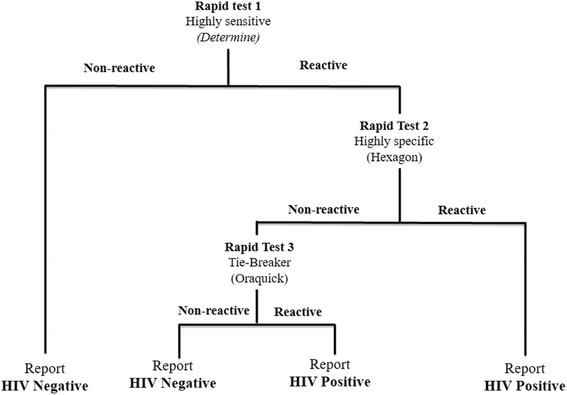
National Algorithm for HIV rapid testing for voluntary and free counseling

A serial algorithm for HIV screening was used in all the PMTCT-SLs and at the NRL, in conformity with the national algorithm for voluntary HIV testing (Fig. 1). Briefly, the first test was Determine HIV1/2 (*Abbott, Minato-ku-Tokyo, Japan*); in case of reactivity of this screening test, Hexagon *(Kora Healthcare, Ireland, United Kingdom)* was used as the confirmatory test. PMTCT-SLs and the NRL received test kits of the same lot that were evaluated through the on-going CDC quality assurance (QA) strengthening program. At the PMTCT-SL, indeterminate HIV results were reported as indeterminate by the respective site, and were then referred to a tiebreaker test at the level of the NRL; however tiebreaker results were not available the same day and so were not recorded on the surveillance form. Specimens tested at the NRL with indeterminate HIV results were subjected to an immediate tiebreaker test, Oraquick *(OraSure Technologies, Inc, Bethlehem, Pennsylvania)*. Residual plasma from all ANC1 was stored at−70 °C in the NRL for quality control or future testing. NRL test result was linked back to the information from the surveillance form through the unique identifiers to create a full data set for analysis.

### Quality assurance of HIV surveillance survey

QA measures were implemented to ensure the quality of surveillance procedures. Staff involved in surveillance (including ANC/PMTCT site clinical and laboratory staff, and central/regional surveillance supervisors) received pre-survey training on the following: data recording in ANC registers and SSS sheets; standard operating procedures for HIV testing; study monitoring, data verification, and sample shipment to NRL. Most PMTCT-SLs were participating in a program for strengthening laboratories capacity on HIV testing, coordinated by the CDC through the use of dry tube specimens; with 100% concordance generally recorded in HIV testing performance. The survey supervisors routinely performed data verification/validation on-site by comparing SSS sheets to ANC registers for accurate data collection. At the central level, data were double entered using CSPro (Census and Survey Processing) version 4 · 1 to minimize errors. Criteria for excluding a sample from the overall analyses were based on unavailable or unreadable or indeterminate results from PMTCT-SL.

### Quality assurance in proficiency testing

The Chantal BIYA International Reference Centre (CIRCB) for research on HIV/AIDS prevention and management (Yaounde, Cameroon) was selected as the NRL following a series of rigorous performance evaluation with external quality control before the survey and then three times during the study, by the CDC-accredited laboratory. CIRCB was then retained as NRL based on the required performance of 100% concordance in all four HIV testing panels (dry tube specimens).

Performance of routine PMTCT-SL in HIV testing was calculated using NRL results as gold standard. Based on conclusive HIV results (i.e. negative or positive) obtained after test 1 and test 2 (*n* = 6056) by both PMTCT-SLs and the NRL, intrinsic (sensitivity and specificity) and extrinsic (positive [PPV] and negative [NPV] predictive values) performances were calculated, as shown in Table 1 [21]. To mitigate potential sources of bias, all PMTCT-SL indeterminate results were excluded from all analyses performed in the survey (both comparisons of individual test results and comparisons of prevalence).

**Table 1:** Calculation of intrinsic and extrinsic performances of HIV testing by PMTCT site laboratories

	National Reference Laboratory (NRL SSS-HIV test)		
PMTCT sites results	HIV+	HIV−	Total
Test +	a	b	a + b
Test−	c	d	c + d
Total	a + c	b + d	a + b + c + d

$$ Reference\;HIV\; Prevalence=100\times \frac{a+c}{a+b+c+d} $$

$$ Sensitivity\kern4.56em =100\times \frac{a}{a+c} $$

$$ Positive\  Predictive\  Value = 100\times \frac{a}{a+b} $$

$$ PMTCT\kern0.24em HIV\kern0.48em  rate\kern1.68em =100\times \frac{a+b}{a+b+c+d} $$

$$ Specificity\kern4.08em =100\times \frac{d}{b+d} $$

$$ Negative\; Predictive\; Value=100\times \frac{d}{c+d} $$

### Ethical considerations

Ethical clearance for the study was obtained from the Cameroon National Ethics Committee. The privacy of consenting pregnant women and their data confidentiality were ensured through a permanent delinking process that does not allow HIV test results to be traced back to any personal identifying information. Data integrity was assured by a double entry and access was restricted for security measures. Onsite HIV test results were offered to pregnant women free of charge, and positive cases were enrolled for PMTCT services according to national guidelines.

### Role of funding source

The Centers of Diseases Control and Prevention (CDC) provided technical support in the study design, result interpretation, and thoroughly reviewed the paper for publication. The local team conducted data collection, data analysis, writing of the report, and initiation of scientific manuscript for publication.

The World Health Organization (WHO) and the United States President’s Emergency Fund for AIDS Relief (PEPFAR) had no direct involvement in the study design, data collection, data analysis, result interpretation, reporting and decision for publishing.

## Results

Nationwide, 93.2% of the HIV-SSS sample size was obtained. The South region did not reach the sample size due to rejection of samples or of data collection sheets for non-conformity.

### Sociodemographic characteristics of the enrolled antenatal care attendees

A total of 6521 ANC1 attendees were enrolled in the study, half (49 · 3%) of them were young (aged 15–24), with up to 75% aged below 30 years (Table 2).

**Table 2:** Distribution of ANC1 attendees according to age and PMTCT site localization (*N* = 6521)

	URBAN		RURAL		Overall	
Age range	Number	%	Number	%	Number	%
<25 years	**1461**	**52** · **41%**	**1756**	**47** · **04%**	**3217**	**49** · **33%**
15–19 years	585	20 · 98%	608	16 · 30%	1193	18 · 29%
19–24 years	876	31 · 43%	1148	30 · 75%	2024	31 · 04%
≥25 years	**1326**	**47** · **59%**	**1978**	**52** · **96%**	**3304**	**50** · **67%**
25–29 years	672	24 · 17%	1004	26 · 84%	1676	25 · 70%
30–34 years	407	14 · 58%	634	16 · 99%	1041	15 · 96%
35–39 years	173	6 · 18%	268	7 · 20%	441	6 · 76%
40–44 years	69	2 · 48%	68	1 · 82%	137	2 · 10%
45–49 years	5	0 · 18%	4	0 · 11%	9	0 · 14%
Total	2787	100 · 00%	3734	100 · 00%	6521	100 · 00%

Legend. numbers and percentages in bold represent the highest sub-populations of study participants

83 · 6% of surveyed pregnant women attended school, with 75 · 3% at a primary or secondary level, against 8 · 3% at a university level (Table 3). Half of the ANC1 attendees were housewives (49 · 7%), whereas 14 · 2% and 33% were students and employees, respectively.

**Table 3:** Distribution of ANC1 attendees according to educational level and site localization (*N* = 6521)

Educational Level	Area of Residence					
	Rural		Urban		Total	
	Number	%	Number	%	Number	%
None	606	21 · 73%	465	12 · 47%	1071	16 · 42%
Primary	1089	39 · 08%	1230	32 · 94%	2319	35 · 56%
Secondary	997	35 · 74%	1595	42 · 73%	2592	39 · 75%
University	95	3 · 45%	444	11 · 85%	539	8 · 27%
Total	2787	100 · 00%	3734	100 · 00%	6521	100 · 00%

### PMTCT HIV testing and SSS NRL results

Overall, the rate of HIV testing acceptance in routine PMTCT was 99 · 1% and all samples were tested both at the PMTCT-SL and at the NRL; with 99 · 6% good laboratory practices for HIV testing at the level of PMTCT-SLs. According to PMCTCT-SL HIV testing results, 47 women samples were tested indeterminate, 417 had missing results; resulting to a total of 6056 conclusive results (positive or negative) at the PMTCT-SLs. Using the tiebreaker (Oraquick), all samples tested at the NRL got a conclusive result (i.e. reported as positive or negative), resulting to a total of 6103 samples (6056 + 47) considered in the final dataset for comparative analysis between PMTCT-SL and the NLR, considering the NRL results as the reference standard.

### Rate of HIV positivity at the PMTCT site laboratories

The general rate of HIV was 6 · 4% (5 · 7% rural versus 6 · 9% urban) based on PMTCT-SLs, with regional disparities, ranging in rural settings from 1 · 9 to 9 · 9% (Far North-South) and in urban settings from 3 · 6 to 12 · 7% (West-Centre). In summary, PMTCT site laboratories generally underestimated HIV prevalence compared to prevalence estimates from the NRL (6 · 46% versus 7 · 18%, respectively Table 4).

**Table 4:** HIV positivity rate at PMTCT site laboratories

Regions	RURAL		URBAN		OVERALL	
	Number of pregnant women tested	HIV positivity from rural PMTCT site laboratory	Number of pregnant women tested	HIV positivity from urban PMTCT site laboratory	Number of pregnant women tested	HIV positivity from site laboratory
Adamawa	287	5 · 57%	403	3 · 72%	690	**4** · **49%**
Centre	199	4 · 02%	308	12 · 66%	507	9 · 27%
East	301	5 · 98%	296	5 · 07%	597	5 · 53%
Far-North	261	1 · 92%	372	7 · 53%	633	5 · 21%
Littoral	254	9 · 06%	393	8 · 65%	647	8 · 81%
North	295	2 · 71%	414	4 · 35%	709	**3** · **67%**
North-West	292	6 · 85%	380	8 · 16%	672	7 · 59%
West	237	5 · 91%	387	3 · 62%	624	**4** · **49%**
South	152	9 · 87%	211	5 · 21%	363	7 · 16%
South-West	282	7 · 09%	379	10 · 82%	661	9 · 23%
Total	2560	5 · 74%	3543	6 · 94%	6103	6 · 44%

NB: in bold are regions with an overall HIV positivity rate <5% from PMTCT laboratories

### Sensitivity and specificity of PMTCT site laboratories in terms of HIV testing

**Fig. 2:**
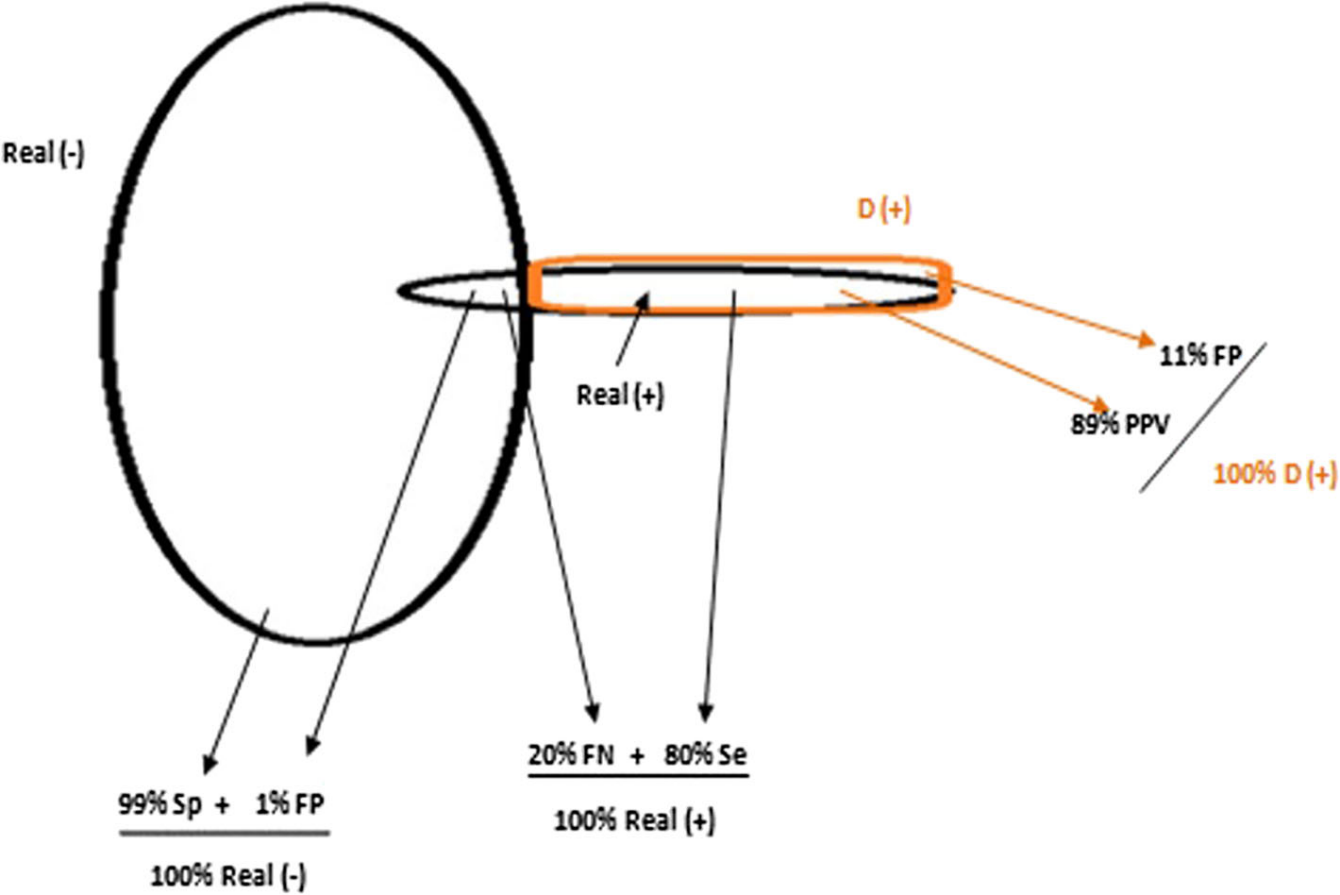
Intersection Analysis of intrinsic and extrinsic performances. Intersection analysis of performance. HIV: Human immunodeficiency virus; NRL: National reference laboratory; PMTCT: prevention of mother to child transmission

Nationwide, the following intrinsic performance of PMTCT-SLs was recorded: a sensitivity of 81 · 2% (from 58 · 8% in the South, to 100% in the West region) and a specificity of 99 · 3% (from 98 · 8% in the South-west and Littoral, to 100% in the Centre region). In terms of geographical location, rural settings had a sensitivity of 76 · 5% and a specificity of 99 · 2%, while urban settings had a sensitivity of 84 · 0% and specificity of 99 · 4%. A regional comparison placed the West region with the highest performance as well as the only region with an acceptable sensitivity (sensitivity of 100% and specificity of 99 · 7%); followed by the Far-North (sensitivity of 93 · 1% and specificity of 99 · 2%) and the North-West (sensitivity of 90 · 2% and specificity of 99 · 3%) regions. In terms of extrinsic performance, performance of PMTCT-SLs reported a national PPV of 88 · 8%, with 84 · 4% in rural and 91 · 5% in urban settings; a national NPV of 98 · 2%, with 98% in rural and 98 · 3% in urban settings. An intersection analysis between intrinsic and extrinsic performances is showed in Fig. 2 (Table 5).

**Table 5:** Sensitivity and Specificity of PMTCT Site Laboratories in HIV testing

Performance Evaluation of PMTCT sites in HIV testing compared to National Reference Laboratory (NRL)															
			Site location												
Region			Rural				Urban				Total				Sensitivity (Se) Specificity (Sp)
			RESULTS FROM THE NATIONAL REFERENCE LABORATORY (NRL)												
	Test Results		Positive	Negative	Indeterminate	Total	Positive	Negative	Indeterminate	Total	Positive	Negative	Indeterminate	Total	
Adamawa	RESULTS PMTCT SITE LABORATORIES	Positive	13	3	0	16	14	1	0	15	27	4	31	31	Se = 81 · 8% Sp = 99 · 4%
		Negative	4	266	1	271	2	384	0	386	6	650	657	657	
		Indeterminate	0	0	0	0	2	0	0	2	2	0	0	2	
		Total	17	269	1	287	18	385	0	403	35	654	1	690	
Centre		Positive	8	0	0	8	39	0	0	39	47	0	0	47	Se = 78 · 3% Sp = 100%
		Negative	8	180	1	189	5	257	0	262	13	437	1	451	
		Indeterminate	1	0	1	2	0	7	0	7	1	7	1	9	
		Total	17	180	2	199	44	264	0	308	61	444	2	507	
East		Positive	16	1	1	18	15	0	0	15	31	1	1	33	Se = 79 · 5% Sp = 99 · 8%
		Negative	7	275	0	282	1	270	3	274	8	545	3	556	
		Indeterminate	0	1	0	1	2	4	1	7	2	5	1	8	
		Total	23	277	1	301	18	274	4	296	41	551	5	597	
Far North		Positive	2	2	1	5	25	3	0	28	27	5	1	33	Se = 93 · 1% Sp = 99 · 2%
		Negative	0	255	0	255	2	340	0	342	2	595	0	597	
		Indeterminate	0	0	1	1	0	0	2	2	0	0	3	3	
		Total	2	257	2	261	27	343	2	372	29	600	4	633	
Littoral		Positive	19	4	0	23	31	3	1	35	50	7	1	58	Se = 79 · 4% Sp = 98 · 8%
		Negative	4	224	1	229	9	339	3	351	13	563	4	580	
		Indeterminate	1	1	0	2	1	5	1	7	2	6	1	9	
		Total	24	229	1	254	41	347	5	393	65	576	6	647	
North		Positive	7	0	1	8	15	3	0	18	22	3	1	26	Se = 71 · 0% Sp = 99 · 6%
		Negative	1	285	1	287	8	384	1	393	9	669	2	680	
		Indeterminate	0	0	0	0	0	2	1	3	0	2	1	3	
		Total	8	285	2	295	23	389	2	414	31	674	4	709	
North-West		Positive	17	3	0	20	29	1	1	31	46	4	1	51	Se = 90 · 2% Sp = 99 · 3%
		Negative	2	267	1	270	3	344	1	348	5	611	2	618	
		Indeterminate	0	2	0	2	0	1	0	1	0	3	0	3	
		Total	19	272	1	292	32	346	2	380	51	618	3	672	
West		Positive	13	1	0	14	13	1	0	14	26	2	0	28	Se = 100% Sp = 99 · 7%
		Negative	0	221	0	221	0	369	2	371	0	590	2	592	
		Indeterminate	0	1	1	2	0	2	0	2	0	3	1	4	
		Total	13	223	1	237	13	372	2	387	26	595	3	624	
South		Positive	11	4	0	15	9	1	1	11	20	5	1	26	Se = 58 · 8% Sp = 99 · 7%
		Negative	7	128	0	135	7	193	0	200	14	321	0	335	
		Indeterminate	0	2	0	2	0	0	0	0	0	2	0	2	
		Total	18	134	0	152	16	194	1	211	34	328	1	363	
South-West		Positive	18	2	0	20	36	5	0	41	54	7	0	61	Se = 83 · 1% Sp = 98 · 8%
		Negative	5	250	4	259	6	329	2	337	11	579	6	596	
		Indeterminate	0	3	0	3	0	0	1	1	0	3	1	4	
		Total	23	255	4	282	42	334	3	379	65	589	7	661	
Total		Positive	124	20	3	147	226	18	3	247	350	38	6	394	
		Negative	38	2351	9	2398	43	3209	12	3264	81	5560	21	5662	
		Indeterminate	2	10	3	15	5	21	6	32	7	31	9	47	
		Total	164	2381	15	2560	274	3248	21	3543	438	5629	36	6103	
Results: Rural, Urban, National			Se = 76 · 5% Sp = 99 · 2%				Se = 84 · 0%; Sp = 99 · 4%				Se = 81 · 2%; Sp = 99 · 3%				

Performances in HIV testing between rural and urban settings showed a statistically significant difference (*p* < 0 · 0001) in the rate of positive-HIV cases obtained from routine-PMTCT rapid testing (6 · 4% positivity rate: 388/6103) against results of the NRL (7 · 1% positivity rate: 431/6103) results. By geographical location, statistically significant differences (*p* < 0 · 0001) were observed when comparing PMTCT rapid testing in rural settings (5 · 6% positivity rate: 144/2560) to that of their corresponding NRL results (6 · 3% positivity rate: 162/6103), and when comparing PMTCT rapid testing in urban settings (6 · 9% positivity rate) to that of the NRL (7 · 1% positivity rate).

### Assessment of indeterminate results both at the PMTCT-SLs and at the NRL

Of the 47 indeterminate results recorded at the PMTCT-SLs after test 1 and test 2, referred to the NRL for tiebreaking, 68 · 0% (32/47) of the samples were from urban vs. 31 · 9% (15/47) from rural settings. At NRL, 7 (14 · 9%) of these indeterminate results were positive, 32 (68 · 0%) were negative, and 9 (19 · 1%) remain indeterminate after test 1 and test 2. Tiebreaker on these 9 persisting indeterminate samples showed all were negative (see Additional file 1: Table S1). Overall, HIV indeterminate results from PMTCT-SLs were 14 · 9% (7/47) positive results vs. and 85 · 1% (40/47) negative.

At the NRL, a total of 36 indeterminate results were recorded after test 1 and test 2. All 36 samples were then tested with the tiebreaker, and revealed a total of 02 (5 · 6%) positive and 34 (94 · 4%) negative (see Additional file 2: Table S2).

As expected, indeterminate results were lower at the NRL (36) vs. PMTCT-SLs (47). In both contexts, indeterminate results were mostly driven by false reactivity of test 1 (94 · 4% at NRL vs. 85 · 1% at PMTCT-SLs).

## Discussion

The overall goal of our study was to assess the feasibility of using routine PMTCT HIV testing data for HIV-SSS among ANC attendees in Cameroon. HIV test results were compared between routine PMTCT HIV testing and testing done at NRL for SSS.

Although the rate of HIV testing acceptance was high and compliance to the national HIV testing algorithm was satisfactory (99 · 6% good laboratory practices), there were poor performances in nine out of the ten regions of Cameroon, translated by significant disparities between HIV positivity rates from routine PMTCT HIV testing and the NRL (*p* = 0 · 002). These data indicate that PMTCT HIV testing will need significant strengthening and quality assurance as Cameroon transitions to using routine PMTCT data for surveillance. The current level of laboratory quality assurance for HIV screening in the PMTCT program remains very low.

Practically, the capacity of routine PMTCT HIV testing to correctly identify HIV-positive pregnant women (sensitivity) was only 81 · 2%, meaning that about 19% of HIV-infected women are wrongly declared HIV seronegative at the PMTCT-SL and are thereby mistakenly excluded from PMTCT care, indicating high risk of poor clinical outcomes and HIV vertical transmission. Regions with lowest sensitivities include the South and littoral regions. Interestingly, the probability of a pregnant woman who received a seropositive HIV result at the PMTCT-SL, to be truly positive (PPV) is 89%, implying that 11% of pregnant women declared HIV seropositive at the PMTCT site laboratory are not infected in principle, those are wrongly declared HIV-positive with unnecessarily enrolled for PMTCT care. As rural settings experienced poorer performance, it is interventions should mostly target these settings that mainly remain at the primary healthcare levels.

In contrast to intrinsic performance, the capacity of each PMTCT-SL in delivering HIV negative results appeared satisfactory (99 · 3% overall). Of note, only one region (West) attained the required target both for the sensitivity and specificity; while the South region recorded the poorest intrinsic performance. Our study indicates an overall tendency of PMTCT data in underestimating the epidemiological burden, which raises important programmatic concerns in a vision of MTCT elimination, since HIV-infected mothers out of care are major reservoirs for onwards vertical transmission [19, 20]. The sensitivity of 81% suggests that about two out of ten HIV-positive (according to NRL testing) ANC1 attendees would be diagnosed negative by PMTCT HIV screening and could transmit HIV vertically. Therefore, the estimated total number of HIV-infected pregnant women who would be missed each year nationwide is 182,588 (ANC1) × 7 · 1% (HIV positivity rate) × 20% (false negative) = 2,593 (HIV-infected women left out of care). Therefore, about 2,000–3,000 HIV-infected pregnant women remain undiagnosed, indicating consistent risks of MTCT, due to inaccurate diagnosis. In spite of the high specificity (99 · 3%), the 0 · 7% pregnant women receiving a false positive HIV result translates into a national estimate of 182,588 (ANC1 per year) × [1–0 · 071] (i.e. 1−positivity rate) × 0 · 7% (false positive) = 1,188 ANC1 with a false positive HIV result every year. Thus, over a thousand of women would be wrongly receiving ART every year, especially with the advent of PMTCT option B+ rollout. This indicates misused resources in an era of poor ART coverage.

For corrective measures, it may be useful to study on-site factors associated with laboratory performance. Well performing sites (i.e. West region) could share best practices to poorly performing sites. The high rate of HIV testing acceptance (favored by the Opt-Out approach in ANC/PMTCT pre-counseling and free screening) excluded possibility of differential HIV prevalence among refusals and accepters, thus limiting bias in PMTCT HIV prevalence estimates [11]. Except for few controversies [12, 21], HIV testing acceptance is often voluntary in African settings [7–11, 13–15, 22–24]. In Mozambique, a sub-Saharan African country with comparable socio-economical realities to Cameroon, using matched PMTCT and SSS results also showed gaps in the quality of PMTCT HIV testing [16]. In addition to previously concordant studies, a major strength of our survey is the large sample size and the high level of national representativeness (regional, rural, urban).

Our major limitation was the difficulties in further exploring discrepant results between the PMTCT-SLs and NRL. Nonetheless, these discrepancies are presumed to be errors in site-level PMTCT testing, since this testing is done by a varied cadre of technicians, in sometimes sub-optimal environmental circumstances, and with limited quality checks (as shown by varying quality systems in place). In contrast, testing at NRL is done by a small group of highly trained, highly supervised technicians in a well-controlled environment, and was subjected to extensive quality control checks. Even if it is concluded that discrepancies were the result of errors at site laboratories, the specific problems were not identified. Errors may occur at the levels of sample labeling/registration (pre-analytical phase), sample testing (analytical) and results interpretation/delivery/recording (post-analytical phase) phases. Identifying these sources of errors would enable relevant recommendations for corrective measures. The poor quality of data in ANC register also stands a potential source of errors, though this was generally solved by supervision. Therefore, the poor concordance between PMTCT-SL and ANC HIV-SSS is likely attributed to poor QA system on-site. Implementing a reliable HIV surveillance system based on routine PMTCT program data warrants continuous training, evaluation, mentorship, and regular supervision of PMTCT sites in LICs like Cameroon.

Interestingly, poor sensitivity of field rapid HIV testing was reported in the South-African PMTCT program using three different rapid tests (94.5% with First Response, 90.2% with Pareekshak, and down to 87.5% with Standard Diagnostic) [25], implying that even with adequate QA system, rapid testing results could be variable, with higher rate of HIV indeterminate results from our urban PMTCT-SLs compared to their rural peers. Of note, indeterminate results were mostly driven by false reactivity of test 1, thus underscoring the necessity of confirming all reactive results after test 1 [25, 26]. This also prompts the need for regular validation, through diagnostic performance evaluation, of rapid tests used in LICs facing similar challenges in the national testing algorithm (against reference standards: fourth generation ELISA, Western Blot and/or molecular diagnostics for confirmation), for an improved evidence-based policy on HIV testing in such settings.

## Conclusion

Despite the high acceptance rate of HIV testing, sensitivity of HIV testing at PMTCT-SL remains poor, indicating the inability to use PMTCT for routine HIV-SSS in Cameroon. These substantial gaps in quality assurance, within routine PMTCT HIV testing pipeline, warrant robust laboratory strengthening measures in an era of transition to using routine PMTCT data for ANC SSS. Even though PMTCT-SL seem highly qualified in screening for uninfected women, sub-optimal performance in detecting HIV-infected pregnant women has important programmatic implications on the effort to eliminate MTCT. Thus, as Cameroon transitions to using PMTCT data for HIV-SSS, strengthening routine HIV testing through training, mentorship, supervision and consistent QA program, is crucial prior to provide robust data for surveillance and for optimal MTCT elimination.

## Additional files

Additional file 1: Table S1. HIV NRL results of indeterminate results at PMTCT-SLs. (DOC 37 kb)
Additional file 2: Table S2. Tiebreaker of the NRL indeterminate results. (DOC 35 kb)

## Abbreviations

AIDS: Acquired immunodeficiency syndrome
ANC1: First antenatal care attendees
CDC: Center for disease control and prevention
CIRCB: Chantal BIYA International Reference Centre for research on HIV/AIDS prevention and management
DHS: Demographic and health surveys
HIV: Human immunodeficiency virus
LIC: Low-income country
NPV: Negative predictive value
NRL: National Reference Laboratory
PEPFAR: United States President’s Emergency Fund for AIDS Relief
PMTCT: Prevention of mother to child transmission
PPV: Positive predictive value
QA: Quality assurance
SL: Site laboratory
SSS: Sentinel surveillance surveys
STI: Sexually transmitted infections
UAT: Unlinked anonymous testing
WHO: World health organization

## References

[1] Institut National de la Statistique, République du Cameroun (2010). Troisième Recensement General de la Population et de l’Habitat (RGPH3).

[2] Institut National de la Statistique, ICF International (2011). Enquête Démographique et de Santé et à indicateurs Multiples du Cameroun 2011. Carverton, Maryland, and Yaoundé, Cameroon.

[3] Organisation Mondiale de la Santé (OMS) (2004). Recommandations pour les enquêtes sérologiques sentinelles concernant le VIH : femmes enceintes et autres groupes.

[4] Institut National de la Statistique (2005). Enquête Démographique et de Santé et à Indicateurs Multiples EDS-MICS 2004.

[5] Institut National de la Statistique (2012). Enquête Démographique et de Santé et à Indicateurs Multiples EDS-MICS 2011.

[6] Comité National de Lutte contre le Sida (2009). Rapport d’enquête de surveillance sentinelle du VIH et de la Syphilis chez les femmes enceintes fréquentant la consultation prénatale en 2009 au Cameroun.

[7] Young PW, Mahomed M, Horth RZ (2013). Routine data from prevention of mother-to-child transmission (PMTCT) HIV testing not yet ready for HIV surveillance in Mozambique: a retrospective analysis of matched test results, February.

[8] Hladik W, Masupu K, Roels T (2005). Prevention of mother-to-child transmission and voluntary counseling and testing program data: what is their utility for HIV surveillance?.

[9] Hladik W, Masupu K, Roels T (2005). Prevention of mother-to-child transmission and voluntary counseling and testing programme data: what is their utility for HIV surveillance? National AIDS Coordinating Agency.

[10] Nicole S, Hladik W, Munyisia E (2003). Can Data from Programs for the Prevention of Mother-to-Child Transmission of HIV be Used for Surveillance in Kenya?.

[11] Chandisarewa W, Stramix-Chibanda L, Chirapa E, et al. Routine off of antenatal HIV testing (“opt-out” approach) to prevent mother-to-child transmission of HIV in urban Zimbabwe. Bull World Health Organ. 2007. 10.2471/BLT.06.035188 PMC2636259 18038074

[12] Kumar R, Virdi NK, Lakshmi PV (2010). Utility of Prevention of Parent-to-Child Transmission (PPTCT). Programme data for HIV surveillance in general population. Indian J Med Res.

[13] Kayibanda JF, Alary M, Bitera R (2011). Use of routine data collected by the prevention of mother-to-child transmission program for HIV surveillance among pregnant women in Rwanda: opportunities and limitation.

[14] Prabhu BD, Seha A, Cambronero I (2012). Identifying Efficiencies in Surveillance: Savings Associated with the Use of PMTCT Program Data for HIV Surveillance in Zanzibar. XVIII International AIDS conference.

[15] Mirkuzie AH, Sisay MM, Hinderaker SG, Moland KM, Mørkve O. Comparing HIV prevalence estimates from prevention of mother-to-child HIV transmission programme and the antenatal HIV surveillance in Addis Ababa of mother-to-child transmission and voluntary counseling and testing programme data: what is their utility for HIV surveillance? City Administration Health Bureau. Addis Ababa, Ethiopia, 2012. 10.1186/1471-2458-12-1113 PMC3533689 23267693

[16] Macauley I, Zekeng L, Mosoko J, et al. HIV prevalence among PMTCT clients and sentinel surveillance in Cameroon. Poster Exhibition: The XV International AIDS Conference: Abstract no. MoPeC3590. 2004.

[17] Marsh KA, Bolu O, Bodika S (2010). Can PMTCT program data replace HIV sentinel surveillance among pregnant women in Africa?. J HIV/AIDS Surveill Epidemiol.

[18] Comité National de Lutte contre le Sida (2012). Vers l’élimination virtuelle de la transmission du VIH de la mère à l’enfant à l’horizon 2015.

[19] Quest Diagnostics. Specimens Handling General Guidelines. © 2000–2014 Quest Diagnostics Incorporated. http://www.questdiagnostics.com/home/physicians/testing-services/specialists/hospitals-lab-staff/specimen-handling/general.html. Accessed 15 Sept 2011

[20] Young PW, Mahomed M, Horth RZ, Shiraishi RW, Jani IV (2013). Routine data from prevention of mother-to-child transmission (PMTCT) HIV testing not yet ready for HIV surveillance in Mozambique: a retrospective analysis of matched test results. BMC Infect Dis.

[21] Statistiques médicales et épidémiologiques. Outils de calcul médico-statistique permettant l’évaluation de la valeur diagnostique d’une méthode de dépistage. www.aly-abbara.com/utilitaires/statistiques/sensibilite_specificite_vpp_vpn.html.

[22] Vidal L, Kuaban C. Sida et tuberculose: la double peine? Institutions, professionnels et sociétés face à la coinfection au Cameroun et au Sénégal. Louvain-La-Neuve, Academia-Bruylant, coll. Espace Afrique, no 9, 378 p., bibliogr., illustr. 2011. URI: http://id.erudit.org/iderudit/1024094ar. Accessed 15 Sept 2012.

[23] PNLS (2009). République démocratique du Congo.

[24] Conseil National de Lutte contre le SIDA, Ministère de la Santé et de la Prévention Médicale, Division de Lutte contre le SIDA/IST, Groupe d’Appui à la Surveillance Epidémiologique, Laboratoire de bactériologie et de Virologie, C.H.U.A.C (2008). Surveillance sentinelle du VIH et de la syphilis chez la femme enceinte.

[25] Black V, Von Mollendorf C, Moyes J, Scott L, Puren A, Stevens W (2009). Poor sensitivity of field rapid HIV testing: implications for mother-to-child transmission programme. BJOG.

[26] Comité National de Lutte contre le Sida (2011). Plan Stratégique National de lutte contre le VIH, le sida et les IST, 2001–2015. Cameroun, Yaoundé.

