# Supplementary material for: Feasibility Study of HIV Sentinel Surveillance using PMTCT data in Cameroon: from Scientific Success to Programmatic Failure
Source: BMC Infect Dis. 2017 Jan 3;17:3. doi: 10.1186/s12879-016-2119-5 (PMC5209823; doi:10.1186/s12879-016-2119-5)
Supplement: Additional file 1: Table S1. — HIV NRL results of indeterminate results at PMTCT-SLs. (DOC 37 kb) [file 12879_2016_2119_MOESM1_ESM.doc]

**Additional file 1: Table S1**: HIV NRL results of indeterminate results at PMTCT-SLs

| Region | **PMTCT site laboratories** | | | **National Reference Laboratory (NRL)** | | | |
| --- | --- | --- | --- | --- | --- | --- | --- |
|  | Total IND | Rural IND | Urban IND | Positive | Negative | IND | Tie-breaker result |
| Adamawa | 2 | 0 | 2 | 2 | 0 | 0 | Not required |
| Centre | 9 | 2 | 7 | 1 | 7 | 1 | NEG |
| East | 8 | 1 | 7 | 2 | 5 | 1 | NEG |
| Far-North | 3 | 1 | 2 | 0 | 0 | 3 | NEG |
| Littoral | 9 | 2 | 7 | 2 | 6 | 1 | NEG |
| North | 3 | 0 | 3 | 0 | 2 | 1 | NEG |
| North-West | 3 | 1 | 2 | 0 | 3 | 0 | Not required |
| West | 4 | 2 | 2 | 0 | 3 | 1 | NEG |
| South | 2 | 2 | 0 | 0 | 2 | 0 | Not required |
| South-West | 4 | 3 | 1 | 0 | 3 | 1 | NEG |
| Total | 47 | 15 | 32 | 7 | 31 | 9 | NEG (all) |

**Legend**: *IND: indeterminate. Of the 47 indeterminate results from the PMTCT-SLs, referred to the NRL for a tiebreaker, 68·0% (32/47) of the samples were from urban vs. 31·9% (15/47) from rural settings. At NRL, 7 (14·9%) of these indeterminate results were positive, 32 (68·0%) were negative, and 9 (19·1%) remain indeterminate after test 1 and test 2. Tiebreaker on these 9 persisting indeterminate samples showed all were* *negative (see supplemental table 1). Overall, HIV indeterminate results from PMTCT-SLs were 14·9% (7/47) positive results vs. and 85·1% (40/47) negative.*
