# Supplementary material for: Feasibility Study of HIV Sentinel Surveillance using PMTCT data in Cameroon: from Scientific Success to Programmatic Failure
Source: BMC Infect Dis. 2017 Jan 3;17:3. doi: 10.1186/s12879-016-2119-5 (PMC5209823; doi:10.1186/s12879-016-2119-5)
Supplement: Additional file 2: Table S2. — Tiebreaker of the NRL indeterminate results. (DOC 35 kb) [file 12879_2016_2119_MOESM2_ESM.doc]

**Additional file 2: Table S2.** Tiebreaker of the NRL indeterminate results

| Region | **Tiebreaker of IND at the National Reference Laboratory (NRL)** | | | **Corresponding results at the PMTCT site laboratories (PMTCT-SLs)** | | |
| --- | --- | --- | --- | --- | --- | --- |
|  | Total IND | Positive | Negative | Positive | Negative | IND |
| Adamawa | 1 | 1 | 0 | 0 | 1 | 0 |
| Centre | 2 | 0 | 2 | 0 | 1 | 1 |
| East | 5 | 0 | 5 | 1 | 3 | 1 |
| Far-North | 4 | 0 | 4 | 1 | 0 | 3 |
| Littoral | 6 | 0 | 6 | 1 | 4 | 1 |
| North | 4 | 1 | 3 | 1 | 2 | 1 |
| North-West | 3 | 0 | 3 | 1 | 2 | 0 |
| West | 3 | 0 | 3 | 0 | 2 | 1 |
| South | 1 | 0 | 1 | 1 | 0 | 0 |
| South-West | 7 | 0 | 7 | 0 | 6 | 1 |
| Total | 36 | 2 | 34 | 6 | 21 | 9 |

**Legend**: *IND: indeterminate. At the NRL total of 36 indeterminate results were recorded after test 1 and test 2. All 36 samples were then tested with the tiebreaker, and revealed a total of 02 (5·6%) positive and 34 (94·4%) negative. Thus, indeterminate results appeared to be mostly driven by false reactivity at test 1.*
